# Supplementary material for: Network-based prediction of the disclosure of ideation about self-harm and suicide in online counseling sessions
Source: Commun Med (Lond). 2022 Dec 6;2:156. doi: 10.1038/s43856-022-00222-4 (PMC9723576; doi:10.1038/s43856-022-00222-4)

Network-based prediction of the disclosure of ideation about self-harm and suicide in online counseling sessions

Zhongzhi Xu, Christian S. Chan*, Qingpeng Zhang, Yucan Xu, Lihong He, Florence Cheung, Jiannan Yang, Evangeline Chan, Jerry Fung, Christy Tsang, and Paul S. F. Yip*

Supplementary Information

Table of Contents

I. Supplementary Methods....2
A. An excerpt of a fictitious counseling session in Cantonese. 2
B. ISS related words and terms 3
C. The bilingual version of Table 1 3
D. The bilingual version of Figure 2....5
II. Supplementary Notes....6
The bilingual version of Figure 4 6

I. Supplementary Methods

A. An excerpt of a fictitious counseling session in Cantonese.

H: I am struggling every day to not jump out of the window. I am suffering and I feel so lonely.
我每日都掙扎緊要吾要跳窗。我真係好辛苦，好孤獨。

C: Hearing this, I am worried about you, L.
聽到 L 咁講，我都擔心你呀…

C: It seems that you feel tormented every day.
每日都好似比病情折磨住咁

H: I feel so muddled. What is the point of life?
好渾渾噩噩，生存究竟有咩意義

H: I want to die so badly. I am suffering and I feel so lonely.
好想死 好辛苦 好孤獨

C: I am here with you, L, you are not alone.
我地都係度陪住 L 呀…你唔孤單

H: I have to wait until October before going back to the clinic for the follow-up consultation. I don't know how to hold on.
十月中先覆診 吾知點捱

H: I want to jump off the building right now. How can I hold on till then?
依家已經好想跳落去 可以點捱

C: Is it possible to make an earlier appointment with the doctor?
會唔會有機會可以同醫生約早 D 個期?

C: Let them know that you are not feeling well lately?

等佢都知道你呢排既狀態唔太好咁

B. ISS related words and terms

Words and phrases used to find *preliminary ISSBs* include 想死 (want to die), 自殺 (suicide), 跳樓 (jump), 離開世界 (leave this world), 死咗 (die), 遺書 (suicide note), 跳落去 (jump from height), 安樂死 (euthanasia), 尋死 (seek death), 去死 (die), 介手 (cut wrist), 界手 (cut wrist), 界刀 (cut), 不想活 (do not wish to live any more), 割脈 (cut vein), 跳樓 (jump from building), 快 D 死 (die quickly), 快 d 死 (die quickly), 自刎 (cut one's throat), 天台 (rooftop), 跌落 (jump off), 企跳 (jump), 自殘 (self-harm), and 鎅 (cut).

C. The bilingual version of Table 1

Table S1. Typical false alarms when relying on keywords matching alone.

False alarm type	Examples
Negation-induced: The action or ideation is negated.	a)我唔係想自殺.<br>It's not that I want to die.<br>b)我冇自殺念頭.<br>I do not have suicidal thoughts.
Subjec***t-induced*:** The action or ideation is about others rather than about the help-seeker.	a)佢話佢好想死但佢唔知點講好.<br>They said they really wanted to die, but didn't know how to talk about it.<br>b)朋友仲要跳樓<br>A friend even died by jumping
Tense-induced: The action or ideation happened in the past and not at present.	a)今年 6 月尾，我跳左樓.<br>At the end of June this year, I jumped of a building.<br>b)我之前一直有好嚴重嘅自殺傾向.<br>I used to have very severe suicidal tendencies.
Other types	syllepsis:<br>a)以前見到英文想死<br>Before, I struggled immensely every time I saw any English

	(Literal: 'Every time I saw any English I'd want to kill myself')
	quoting others:<br>a)話我扮曬嘢自殺係 attention seeking.<br>They said I was only pretending to be suicidal because I was attention seeking.<br>b)叫我點解唔去跳樓死左<br>…Telling me why not just jump and go kill myself.
	dream:<br>a)佢地次次嘈完個晚都會發惡夢但係次次都會跌落樓.<br>Every time they argue I get nightmares, and in each one I fall off a building<br>b)有幾晚發惡夢自己系天台跳落去.<br>There were several nights where I had nightmares of jumping off a rooftop

D. The bilingual version of Figure 2

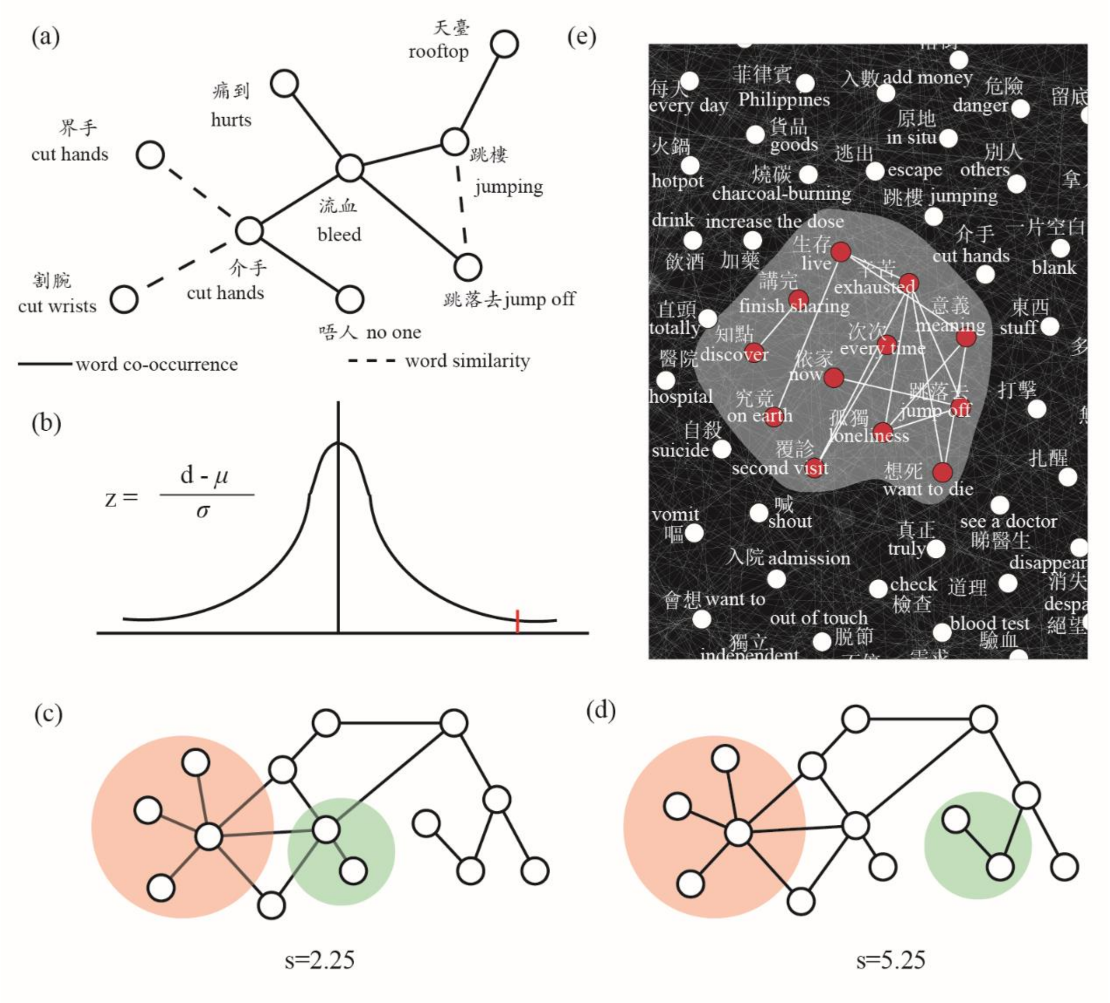

II. Supplementary Notes

The bilingual version of Figure 4

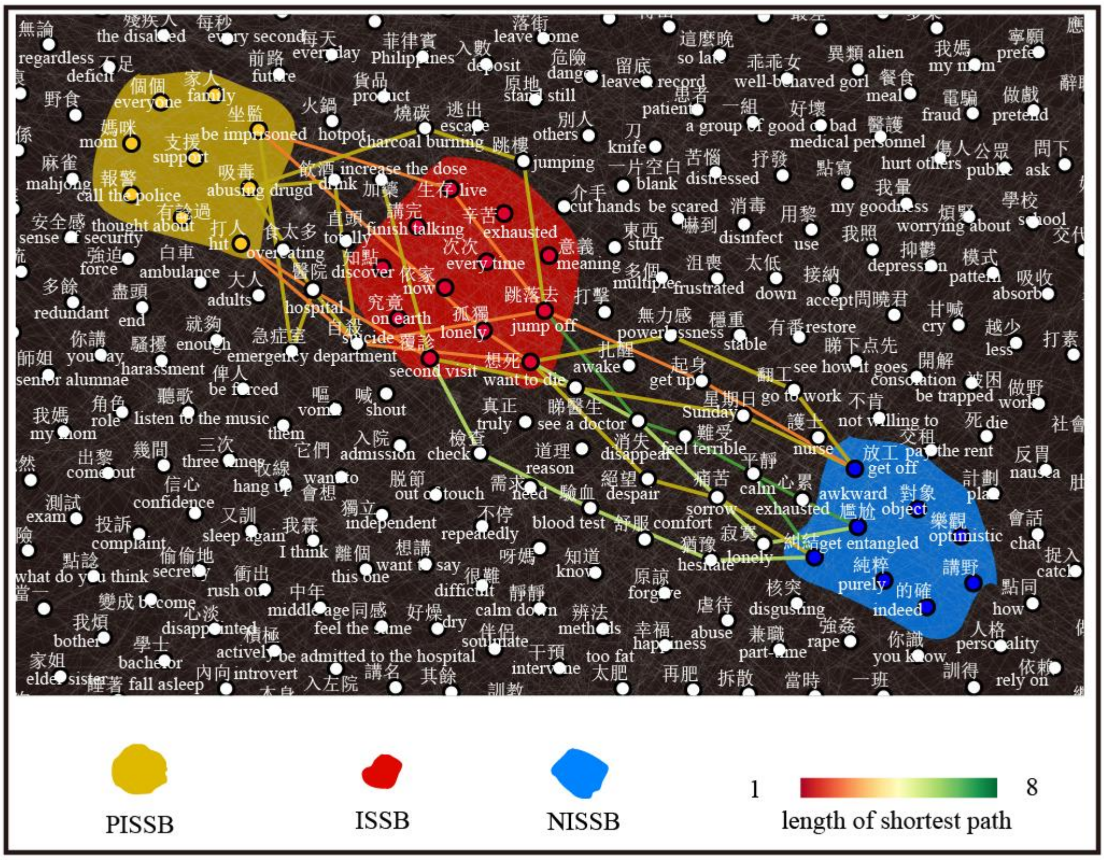

Supplement: Supplementary file 2 — Supplementary Material [file 43856_2022_222_MOESM2_ESM.pdf]
